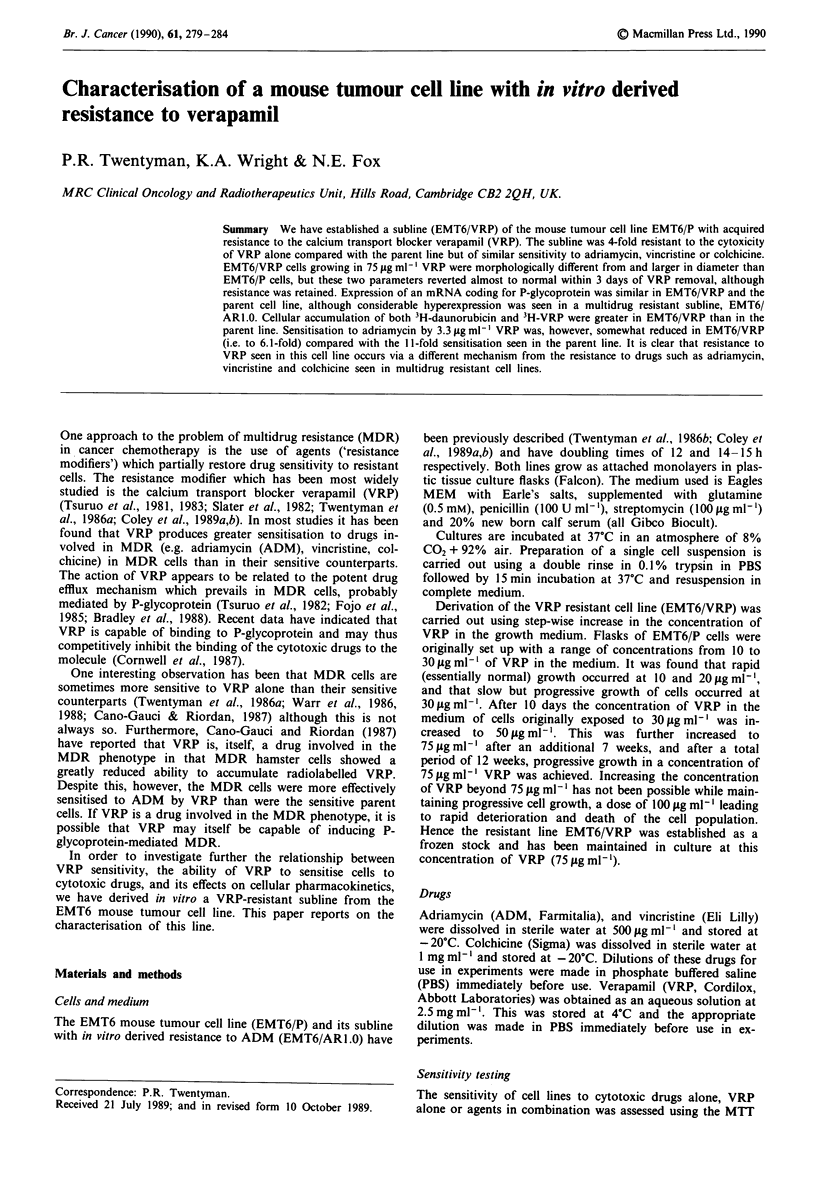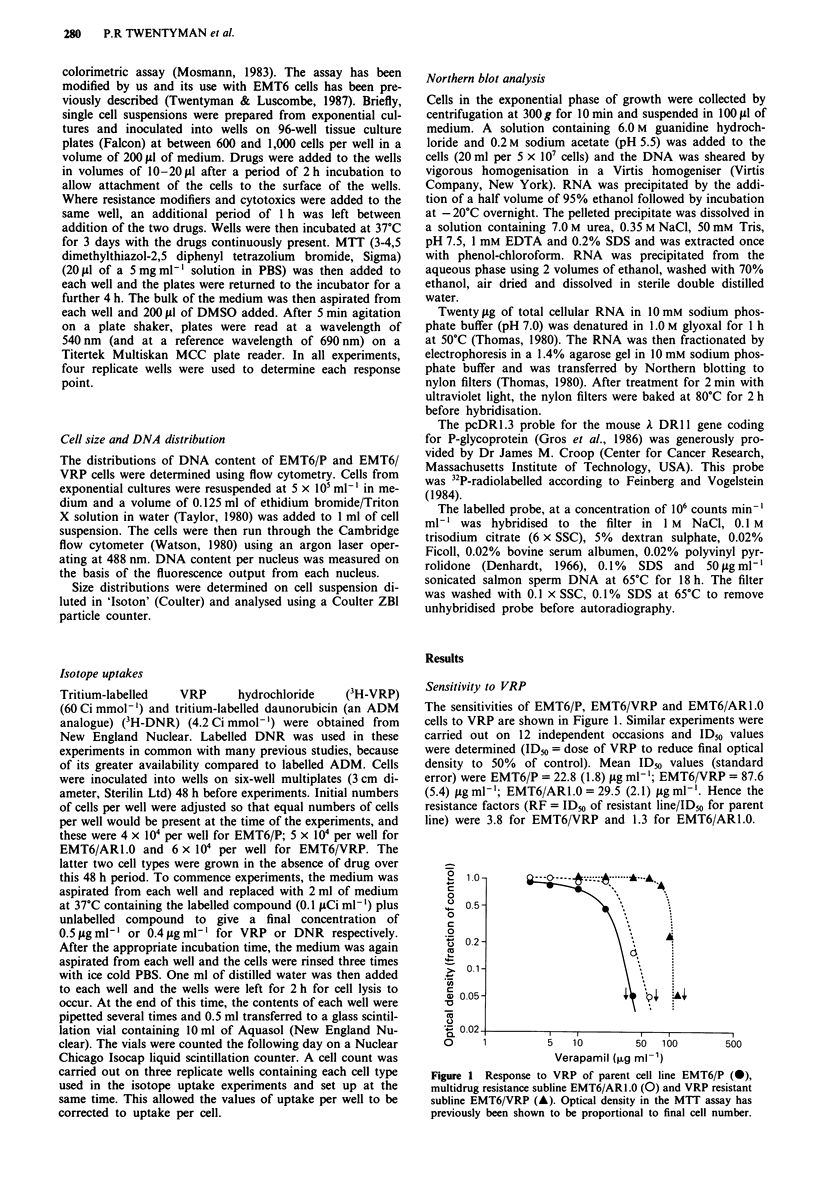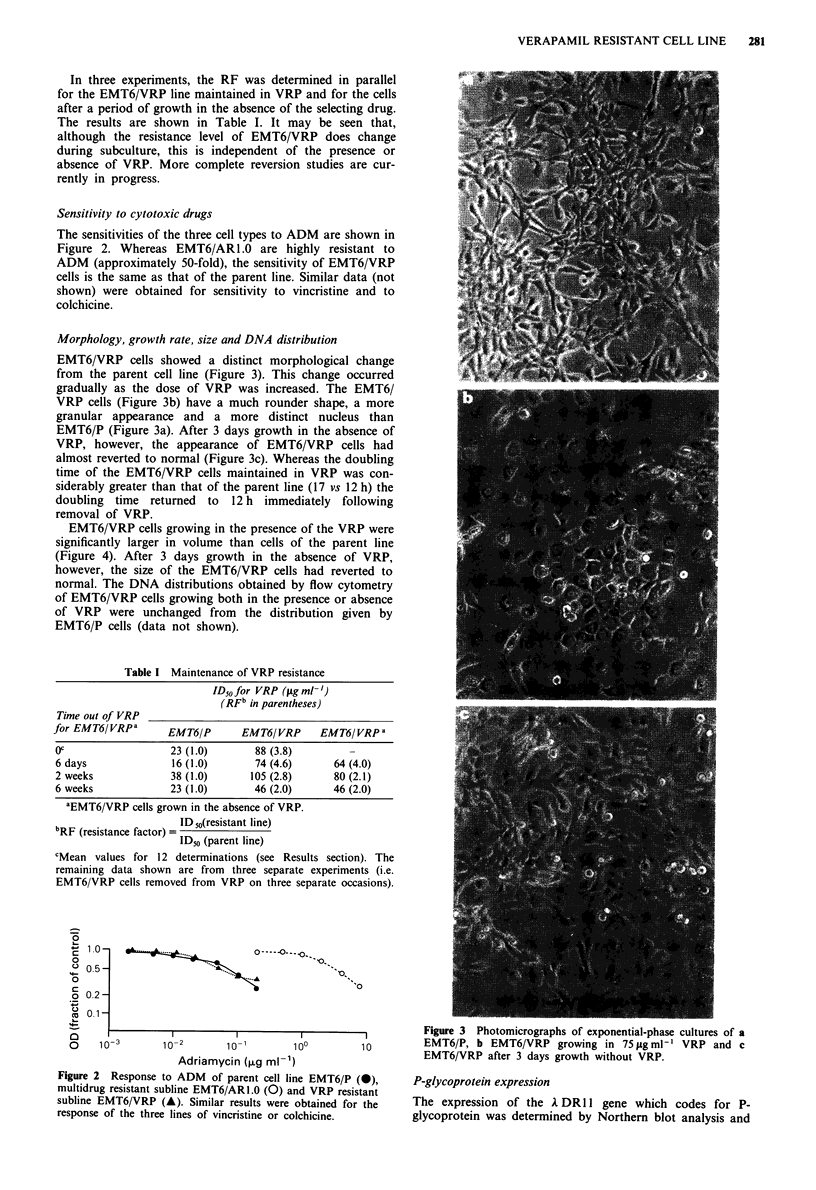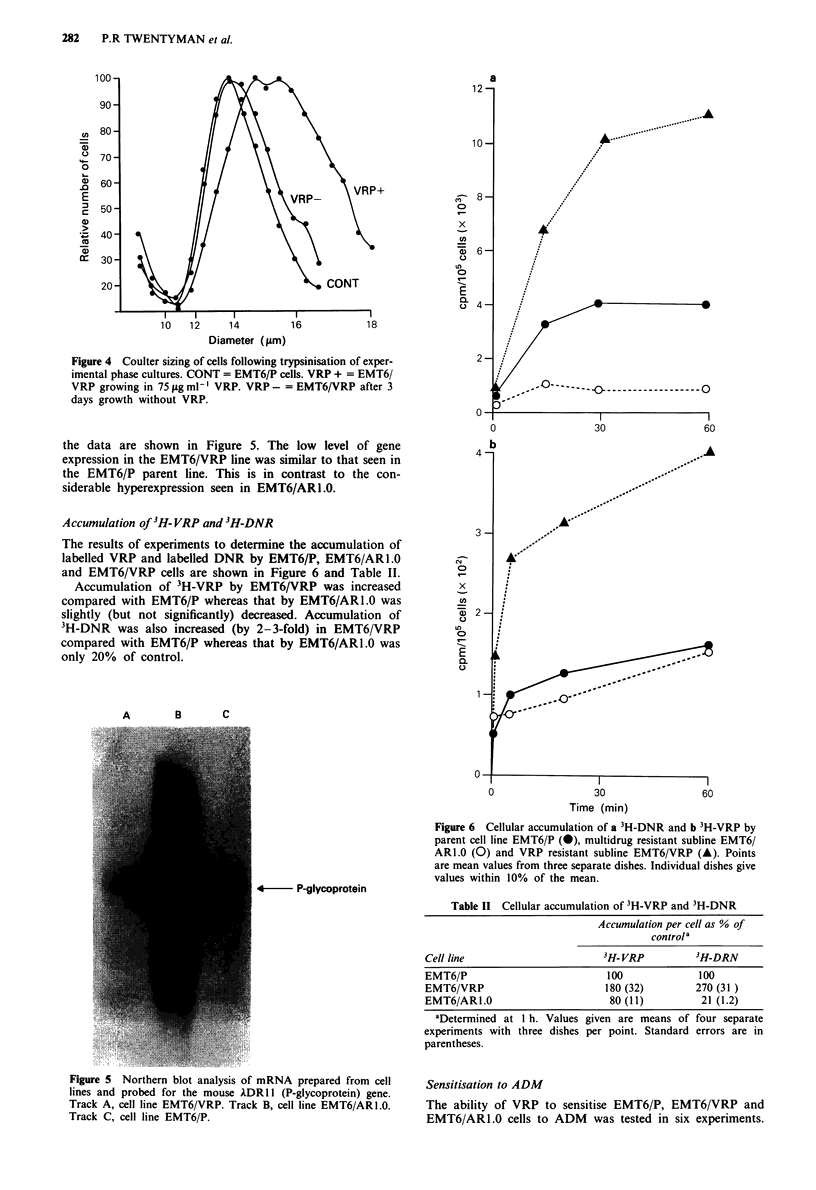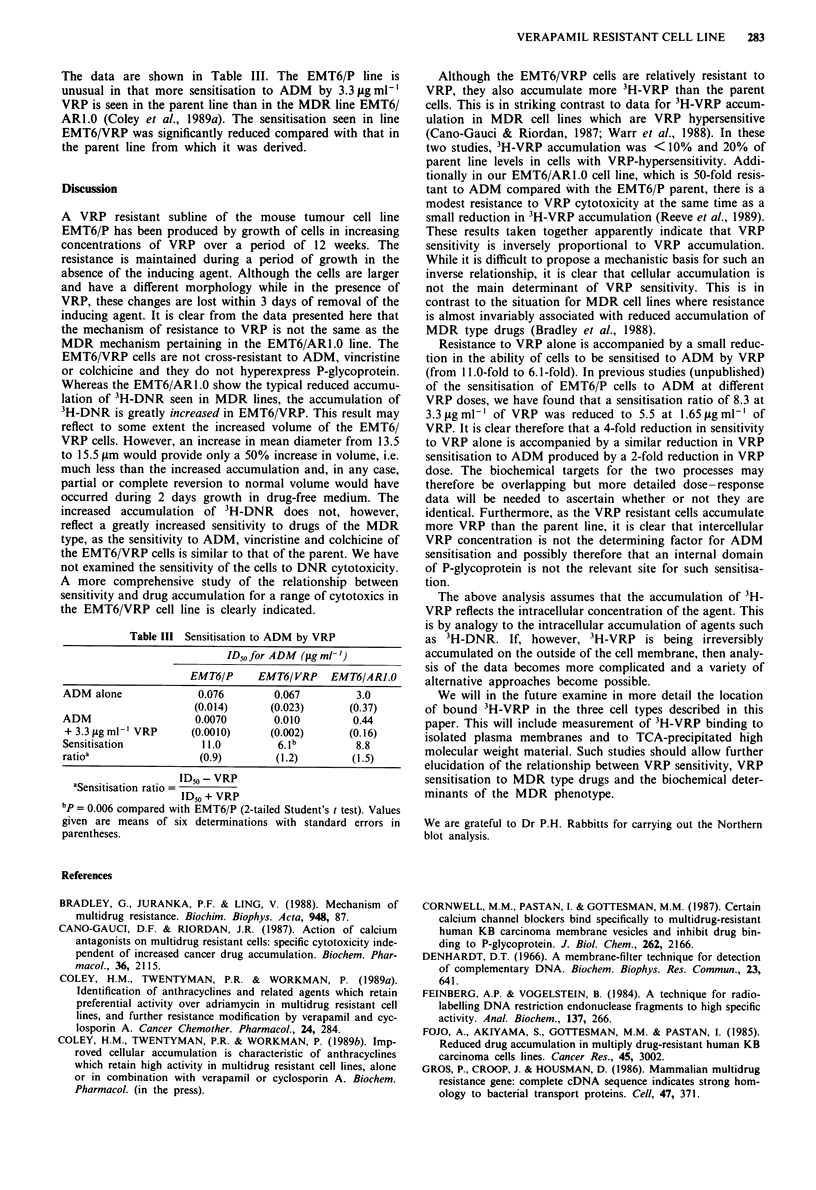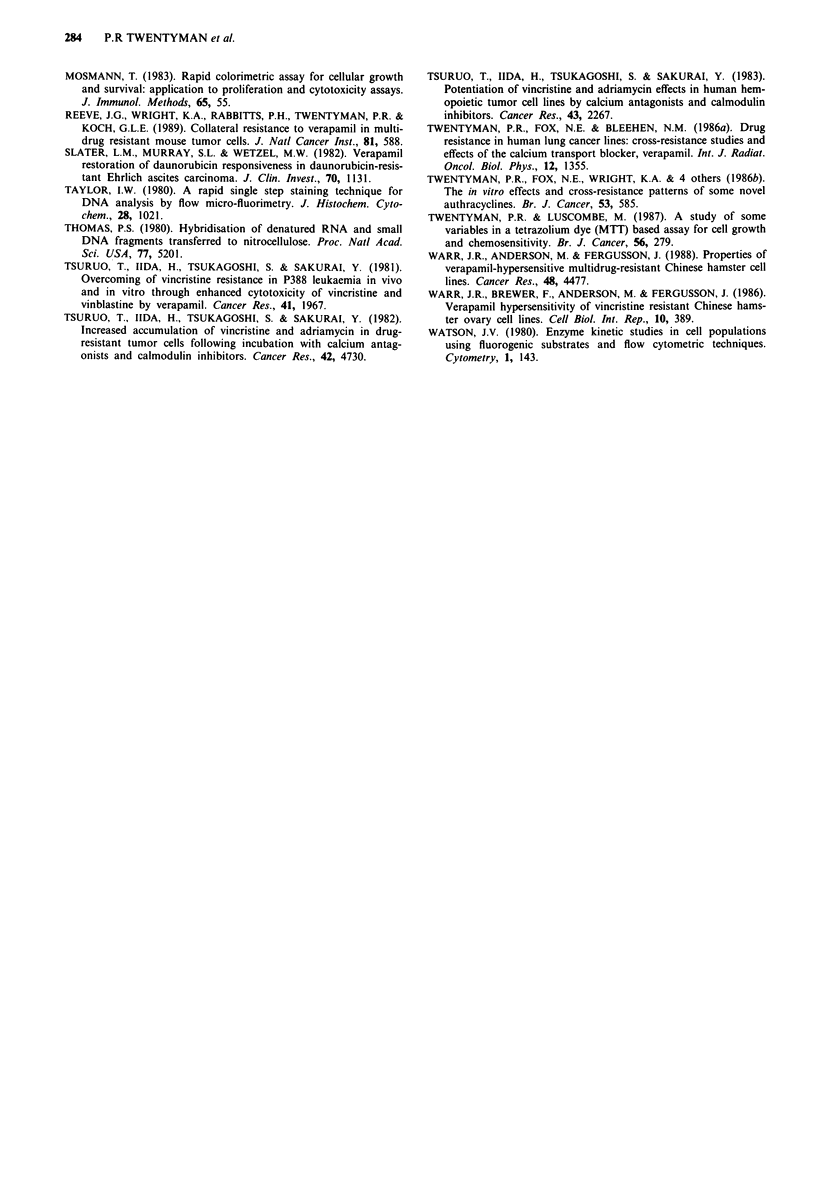# Characterisation of a mouse tumour cell line with in vitro derived resistance to verapamil.

**DOI:** 10.1038/bjc.1990.52

**Published:** 1990-02

**Authors:** P. R. Twentyman, K. A. Wright, N. E. Fox

**Affiliations:** MRC Clinical Oncology and Radiotherapeutics Unit, Cambridge, UK.

## Abstract

**Images:**


Br. J. Cancer (1990), 61, 279–284 © Macmillan Press Ltd., 1990

### Characterisation of a mouse tumour cell line with *in vitro* derived resistance to verapamil

P.R. Twentyman, K.A. Wright & N.E. Fox

*MRC Clinical Oncology and Radiotherapeutics Unit, Hills Road, Cambridge CB2 2QH, UK.*

**Summary** We have established a subline (EMT6/VRP) of the mouse tumour cell line EMT6/P with acquired resistance to the calcium transport blocker verapamil (VRP). The subline was 4-fold resistant to the cytoxicity of VRP alone compared with the parent line but of similar sensitivity to adriamycin, vincristine or colchicine. EMT6/VRP cells growing in 75 μg ml$^{-1}$ VRP were morphologically different from and larger in diameter than EMT6/P cells, but these two parameters reverted almost to normal within 3 days of VRP removal, although resistance was retained. Expression of an mRNA coding for P-glycoprotein was similar in EMT6/VRP and the parent cell line, although considerable hyperexpression was seen in a multidrug resistant subline, EMT6/AR1.0. Cellular accumulation of both $^{3}$H-daunorubicin and $^{3}$H-VRP were greater in EMT6/VRP than in the parent line. Sensitisation to adriamycin by 3.3 μg ml$^{-1}$ VRP was, however, somewhat reduced in EMT6/VRP (i.e. to 6.1-fold) compared with the 11-fold sensitisation seen in the parent line. It is clear that resistance to VRP seen in this cell line occurs via a different mechanism from the resistance to drugs such as adriamycin, vincristine and colchicine seen in multidrug resistant cell lines.

One approach to the problem of multidrug resistance (MDR) in cancer chemotherapy is the use of agents ('resistance modifiers') which partially restore drug sensitivity to resistant cells. The resistance modifier which has been most widely studied is the calcium transport blocker verapamil (VRP) (Tsuruo *et al.*, 1981, 1983; Slater *et al.*, 1982; Twentyman *et al.*, 1986*a*; Coley *et al.*, 1989*a*,*b*). In most studies it has been found that VRP produces greater sensitisation to drugs involved in MDR (e.g. adriamycin (ADM), vincristine, colchicine) in MDR cells than in their sensitive counterparts. The action of VRP appears to be related to the potent drug efflux mechanism which prevails in MDR cells, probably mediated by P-glycoprotein (Tsuruo *et al.*, 1982; Fojo *et al.*, 1985; Bradley *et al.*, 1988). Recent data have indicated that VRP is capable of binding to P-glycoprotein and may thus competitively inhibit the binding of the cytotoxic drugs to the molecule (Cornwell *et al.*, 1987).

One interesting observation has been that MDR cells are sometimes more sensitive to VRP alone than their sensitive counterparts (Twentyman *et al.*, 1986*a*; Warr *et al.*, 1986, 1988; Cano-Gauci & Riordan, 1987) although this is not always so. Furthermore, Cano-Gauci and Riordan (1987) have reported that VRP is, itself, a drug involved in the MDR phenotype in that MDR hamster cells showed a greatly reduced ability to accumulate radiolabelled VRP. Despite this, however, the MDR cells were more effectively sensitised to ADM by VRP than were the sensitive parent cells. If VRP is a drug involved in the MDR phenotype, it is possible that VRP may itself be capable of inducing P-glycoprotein-mediated MDR.

In order to investigate further the relationship between VRP sensitivity, the ability of VRP to sensitise cells to cytotoxic drugs, and its effects on cellular pharmacokinetics, we have derived *in vitro* a VRP-resistant subline from the EMT6 mouse tumour cell line. This paper reports on the characterisation of this line.

### Materials and methods

### Cells and medium

The EMT6 mouse tumour cell line (EMT6/P) and its subline with *in vitro* derived resistance to ADM (EMT6/AR1.0) have been previously described (Twentyman *et al.*, 1986*b*; Coley *et al.*, 1989*a*,*b*) and have doubling times of 12 and 14–15 h respectively. Both lines grow as attached monolayers in plastic tissue culture flasks (Falcon). The medium used is Eagles MEM with Earle's salts, supplemented with glutamine (0.5 mM), penicillin (100 U ml$^{-1}$), streptomycin (100 μg ml$^{-1}$) and 20% new born calf serum (all Gibco Biocult).

Cultures are incubated at 37°C in an atmosphere of 8% $CO_2$ + 92% air. Preparation of a single cell suspension is carried out using a double rinse in 0.1% trypsin in PBS followed by 15 min incubation at 37°C and resuspension in complete medium.

Derivation of the VRP resistant cell line (EMT6/VRP) was carried out using step-wise increase in the concentration of VRP in the growth medium. Flasks of EMT6/P cells were originally set up with a range of concentrations from 10 to 30 μg ml$^{-1}$ of VRP in the medium. It was found that rapid (essentially normal) growth occurred at 10 and 20 μg ml$^{-1}$, and that slow but progressive growth of cells occurred at 30 μg ml$^{-1}$. After 10 days the concentration of VRP in the medium of cells originally exposed to 30 μg ml$^{-1}$ was increased to 50 μg ml$^{-1}$. This was further increased to 75 μg ml$^{-1}$ after an additional 7 weeks, and after a total period of 12 weeks, progressive growth in a concentration of 75 μg ml$^{-1}$ VRP was achieved. Increasing the concentration of VRP beyond 75 μg ml$^{-1}$ has not been possible while maintaining progressive cell growth, a dose of 100 μg ml$^{-1}$ leading to rapid deterioration and death of the cell population. Hence the resistant line EMT6/VRP was established as a frozen stock and has been maintained in culture at this concentration of VRP (75 μg ml$^{-1}$).

### Drugs

Adriamycin (ADM, Farmitalia), and vincristine (Eli Lilly) were dissolved in sterile water at 500 μg ml$^{-1}$ and stored at −20°C. Colchicine (Sigma) was dissolved in sterile water at 1 mg ml$^{-1}$ and stored at −20°C. Dilutions of these drugs for use in experiments were made in phosphate buffered saline (PBS) immediately before use. Verapamil (VRP, Cordilox, Abbott Laboratories) was obtained as an aqueous solution at 2.5 mg ml$^{-1}$. This was stored at 4°C and the appropriate dilution was made in PBS immediately before use in experiments.

### Sensitivity testing

The sensitivity of cell lines to cytotoxic drugs alone, VRP alone or agents in combination was assessed using the MTT

Correspondence: P.R. Twentyman.
Received 21 July 1989; and in revised form 10 October 1989.

colorimetric assay (Mosmann, 1983). The assay has been modified by us and its use with EMT6 cells has been previously described (Twentyman & Luscombe, 1987). Briefly, single cell suspensions were prepared from exponential cultures and inoculated into wells on 96-well tissue culture plates (Falcon) at between 600 and 1,000 cells per well in a volume of 200 μl of medium. Drugs were added to the wells in volumes of 10–20 μl after a period of 2 h incubation to allow attachment of the cells to the surface of the wells. Where resistance modifiers and cytotoxics were added to the same well, an additional period of 1 h was left between addition of the two drugs. Wells were then incubated at 37°C for 3 days with the drugs continuously present. MTT (3-4,5 dimethylthiazol-2,5 diphenyl tetrazolium bromide, Sigma) (20 μl of a 5 mg ml$^{-1}$ solution in PBS) was then added to each well and the plates were returned to the incubator for a further 4 h. The bulk of the medium was then aspirated from each well and 200 μl of DMSO added. After 5 min agitation on a plate shaker, plates were read at a wavelength of 540 nm (and at a reference wavelength of 690 nm) on a Titertek Multiskan MCC plate reader. In all experiments, four replicate wells were used to determine each response point.

*Cell size and DNA distribution*

The distributions of DNA content of EMT6/P and EMT6/VRP cells were determined using flow cytometry. Cells from exponential cultures were resuspended at $5 \times 10^5$ ml$^{-1}$ in medium and a volume of 0.125 ml of ethidium bromide/Triton X solution in water (Taylor, 1980) was added to 1 ml of cell suspension. The cells were then run through the Cambridge flow cytometer (Watson, 1980) using an argon laser operating at 488 nm. DNA content per nucleus was measured on the basis of the fluorescence output from each nucleus.

Size distributions were determined on cell suspension diluted in 'Isoton' (Coulter) and analysed using a Coulter ZBI particle counter.

*Isotope uptakes*

Tritium-labelled VRP hydrochloride ($^3$H-VRP) (60 Ci mmol$^{-1}$) and tritium-labelled daunorubicin (an ADM analogue) ($^3$H-DNR) (4.2 Ci mmol$^{-1}$) were obtained from New England Nuclear. Labelled DNR was used in these experiments in common with many previous studies, because of its greater availability compared to labelled ADM. Cells were inoculated into wells on six-well multiplates (3 cm diameter, Sterilin Ltd) 48 h before experiments. Initial numbers of cells per well were adjusted so that equal numbers of cells per well would be present at the time of the experiments, and these were $4 \times 10^4$ per well for EMT6/P; $5 \times 10^4$ per well for EMT6/AR1.0 and $6 \times 10^4$ per well for EMT6/VRP. The latter two cell types were grown in the absence of drug over this 48 h period. To commence experiments, the medium was aspirated from each well and replaced with 2 ml of medium at 37°C containing the labelled compound (0.1 μCi ml$^{-1}$) plus unlabelled compound to give a final concentration of 0.5 μg ml$^{-1}$ or 0.4 μg ml$^{-1}$ for VRP or DNR respectively. After the appropriate incubation time, the medium was again aspirated from each well and the cells were rinsed three times with ice cold PBS. One ml of distilled water was then added to each well and the wells were left for 2 h for cell lysis to occur. At the end of this time, the contents of each well were pipetted several times and 0.5 ml transferred to a glass scintillation vial containing 10 ml of Aquasol (New England Nuclear). The vials were counted the following day on a Nuclear Chicago Isocap liquid scintillation counter. A cell count was carried out on three replicate wells containing each cell type used in the isotope uptake experiments and set up at the same time. This allowed the values of uptake per well to be corrected to uptake per cell.

*Northern blot analysis*

Cells in the exponential phase of growth were collected by centrifugation at 300 *g* for 10 min and suspended in 100 μl of medium. A solution containing 6.0 M guanidine hydrochloride and 0.2 M sodium acetate (pH 5.5) was added to the cells (20 ml per $5 \times 10^7$ cells) and the DNA was sheared by vigorous homogenisation in a Virtis homogeniser (Virtis Company, New York). RNA was precipitated by the addition of a half volume of 95% ethanol followed by incubation at −20°C overnight. The pelleted precipitate was dissolved in a solution containing 7.0 M urea, 0.35 M NaCl, 50 mM Tris, pH 7.5, 1 mM EDTA and 0.2% SDS and was extracted once with phenol-chloroform. RNA was precipitated from the aqueous phase using 2 volumes of ethanol, washed with 70% ethanol, air dried and dissolved in sterile double distilled water.

Twenty μg of total cellular RNA in 10 mM sodium phosphate buffer (pH 7.0) was denatured in 1.0 M glyoxal for 1 h at 50°C (Thomas, 1980). The RNA was then fractionated by electrophoresis in a 1.4% agarose gel in 10 mM sodium phosphate buffer and was transferred by Northern blotting to nylon filters (Thomas, 1980). After treatment for 2 min with ultraviolet light, the nylon filters were baked at 80°C for 2 h before hybridisation.

The pcDR1.3 proble for the mouse λ DR11 gene coding for P-glycoprotein (Gros *et al.*, 1986) was generously provided by Dr James M. Croop (Center for Cancer Research, Massachusetts Institute of Technology, USA). This probe was $^{32}$P-radiolabelled according to Feinberg and Vogelstein (1984).

The labelled probe, at a concentration of $10^6$ counts min$^{-1}$ ml$^{-1}$ was hybridised to the filter in 1 M NaCl, 0.1 M trisodium citrate (6 × SSC), 5% dextran sulphate, 0.02% Ficoll, 0.02% bovine serum albumen, 0.02% polyvinyl pyrrolidone (Denhardt, 1966), 0.1% SDS and 50 μg ml$^{-1}$ sonicated salmon sperm DNA at 65°C for 18 h. The filter was washed with 0.1 × SSC, 0.1% SDS at 65°C to remove unhybridised probe before autoradiography.

### Results

*Sensitivity to VRP*

The sensitivities of EMT6/P, EMT6/VRP and EMT6/AR1.0 cells to VRP are shown in Figure 1. Similar experiments were carried out on 12 independent occasions and $ID_{50}$ values were determined ($ID_{50}$ = dose of VRP to reduce final optical density to 50% of control). Mean $ID_{50}$ values (standard error) were EMT6/P = 22.8 (1.8) μg ml$^{-1}$; EMT6/VRP = 87.6 (5.4) μg ml$^{-1}$; EMT6/AR1.0 = 29.5 (2.1) μg ml$^{-1}$. Hence the resistance factors (RF = $ID_{50}$ of resistant line/$ID_{50}$ for parent line) were 3.8 for EMT6/VRP and 1.3 for EMT6/AR1.0.

**Figure 1** Response to VRP of parent cell line EMT6/P (●), multidrug resistance subline EMT6/AR1.0 (○) and VRP resistant subline EMT6/VRP (▲). Optical density in the MTT assay has previously been shown to be proportional to final cell number.

In three experiments, the RF was determined in parallel for the EMT6/VRP line maintained in VRP and for the cells after a period of growth in the absence of the selecting drug. The results are shown in Table I. It may be seen that, although the resistance level of EMT6/VRP does change during subculture, this is independent of the presence or absence of VRP. More complete reversion studies are currently in progress.

*Sensitivity to cytotoxic drugs*

The sensitivities of the three cell types to ADM are shown in Figure 2. Whereas EMT6/AR1.0 are highly resistant to ADM (approximately 50-fold), the sensitivity of EMT6/VRP cells is the same as that of the parent line. Similar data (not shown) were obtained for sensitivity to vincristine and to colchicine.

*Morphology, growth rate, size and DNA distribution*

EMT6/VRP cells showed a distinct morphological change from the parent cell line (Figure 3). This change occurred gradually as the dose of VRP was increased. The EMT6/VRP cells (Figure 3b) have a much rounder shape, a more granular appearance and a more distinct nucleus than EMT6/P (Figure 3a). After 3 days growth in the absence of VRP, however, the appearance of EMT6/VRP cells had almost reverted to normal (Figure 3c). Whereas the doubling time of the EMT6/VRP cells maintained in VRP was considerably greater than that of the parent line (17 *vs* 12 h) the doubling time returned to 12 h immediately following removal of VRP.

EMT6/VRP cells growing in the presence of the VRP were significantly larger in volume than cells of the parent line (Figure 4). After 3 days growth in the absence of VRP, however, the size of the EMT6/VRP cells had reverted to normal. The DNA distributions obtained by flow cytometry of EMT6/VRP cells growing both in the presence or absence of VRP were unchanged from the distribution given by EMT6/P cells (data not shown).

**Table I** Maintenance of VRP resistance

| Time out of VRP for EMT6/VRP[a] | $ID_{50}$ for VRP ($\mu g\ ml^{-1}$) (RF[b] in parentheses) EMT6/P | EMT6/VRP | EMT6/VRP[a] |
|---|---|---|---|
| 0[c] | 23 (1.0) | 88 (3.8) | – |
| 6 days | 16 (1.0) | 74 (4.6) | 64 (4.0) |
| 2 weeks | 38 (1.0) | 105 (2.8) | 80 (2.1) |
| 6 weeks | 23 (1.0) | 46 (2.0) | 46 (2.0) |

[a]EMT6/VRP cells grown in the absence of VRP.

[b]RF (resistance factor) = $\frac{ID_{50}(\text{resistant line})}{ID_{50}\ (\text{parent line})}$

[c]Mean values for 12 determinations (see Results section). The remaining data shown are from three separate experiments (i.e. EMT6/VRP cells removed from VRP on three separate occasions).

**Figure 2** Response to ADM of parent cell line EMT6/P (●), multidrug resistant subline EMT6/AR1.0 (○) and VRP resistant subline EMT6/VRP (▲). Similar results were obtained for the response of the three lines of vincristine or colchicine.

**Figure 3** Photomicrographs of exponential-phase cultures of **a** EMT6/P, **b** EMT6/VRP growing in 75 μg ml$^{-1}$ VRP and **c** EMT6/VRP after 3 days growth without VRP.

*P-glycoprotein expression*

The expression of the λ DR11 gene which codes for P-glycoprotein was determined by Northern blot analysis and

**Figure 4** Coulter sizing of cells following trypsinisation of experimental phase cultures. CONT = EMT6/P cells. VRP + = EMT6/VRP growing in 75 μg ml$^{-1}$ VRP. VRP − = EMT6/VRP after 3 days growth without VRP.

the data are shown in Figure 5. The low level of gene expression in the EMT6/VRP line was similar to that seen in the EMT6/P parent line. This is in contrast to the considerable hyperexpression seen in EMT6/AR1.0.

*Accumulation of $^3$H-VRP and $^3$H-DNR*

The results of experiments to determine the accumulation of labelled VRP and labelled DNR by EMT6/P, EMT6/AR1.0 and EMT6/VRP cells are shown in Figure 6 and Table II.

Accumulation of $^3$H-VRP by EMT6/VRP was increased compared with EMT6/P whereas that by EMT6/AR1.0 was slightly (but not significantly) decreased. Accumulation of $^3$H-DNR was also increased (by 2–3-fold) in EMT6/VRP compared with EMT6/P whereas that by EMT6/AR1.0 was only 20% of control.

**Figure 5** Northern blot analysis of mRNA prepared from cell lines and probed for the mouse λDR11 (P-glycoprotein) gene. Track A, cell line EMT6/VRP. Track B, cell line EMT6/AR1.0. Track C, cell line EMT6/P.

**Figure 6** Cellular accumulation of **a** $^3$H-DNR and **b** $^3$H-VRP by parent cell line EMT6/P (●), multidrug resistant subline EMT6/AR1.0 (○) and VRP resistant subline EMT6/VRP (▲). Points are mean values from three separate dishes. Individual dishes give values within 10% of the mean.

**Table II** Cellular accumulation of $^3$H-VRP and $^3$H-DNR

| | *Accumulation per cell as % of control*[a] | |
|---|---|---|
| *Cell line* | *$^3$H-VRP* | *$^3$H-DRN* |
| EMT6/P | 100 | 100 |
| EMT6/VRP | 180 (32) | 270 (31 ) |
| EMT6/AR1.0 | 80 (11) | 21 (1.2) |

[a]Determined at 1 h. Values given are means of four separate experiments with three dishes per point. Standard errors are in parentheses.

*Sensitisation to ADM*

The ability of VRP to sensitise EMT6/P, EMT6/VRP and EMT6/AR1.0 cells to ADM was tested in six experiments.

The data are shown in Table III. The EMT6/P line is unusual in that more sensitisation to ADM by 3.3 μg ml$^{-1}$ VRP is seen in the parent line than in the MDR line EMT6/AR1.0 (Coley *et al.*, 1989*a*). The sensitisation seen in line EMT6/VRP was significantly reduced compared with that in the parent line from which it was derived.

### Discussion

A VRP resistant subline of the mouse tumour cell line EMT6/P has been produced by growth of cells in increasing concentrations of VRP over a period of 12 weeks. The resistance is maintained during a period of growth in the absence of the inducing agent. Although the cells are larger and have a different morphology while in the presence of VRP, these changes are lost within 3 days of removal of the inducing agent. It is clear from the data presented here that the mechanism of resistance to VRP is not the same as the MDR mechanism pertaining in the EMT6/AR1.0 line. The EMT6/VRP cells are not cross-resistant to ADM, vincristine or colchicine and they do not hyperexpress P-glycoprotein. Whereas the EMT6/AR1.0 show the typical reduced accumulation of $^3$H-DNR seen in MDR lines, the accumulation of $^3$H-DNR is greatly *increased* in EMT6/VRP. This result may reflect to some extent the increased volume of the EMT6/VRP cells. However, an increase in mean diameter from 13.5 to 15.5 μm would provide only a 50% increase in volume, i.e. much less than the increased accumulation and, in any case, partial or complete reversion to normal volume would have occurred during 2 days growth in drug-free medium. The increased accumulation of $^3$H-DNR does not, however, reflect a greatly increased sensitivity to drugs of the MDR type, as the sensitivity to ADM, vincristine and colchicine of the EMT6/VRP cells is similar to that of the parent. We have not examined the sensitivity of the cells to DNR cytotoxicity. A more comprehensive study of the relationship between sensitivity and drug accumulation for a range of cytotoxics in the EMT6/VRP cell line is clearly indicated.

**Table III** Sensitisation to ADM by VRP

| | *ID$_{50}$ for ADM (μg ml$^{-1}$)* | | |
|---|---|---|---|
| | *EMT6/P* | *EMT6/VRP* | *EMT6/AR1.0* |
| ADM alone | 0.076 | 0.067 | 3.0 |
| | (0.014) | (0.023) | (0.37) |
| ADM | 0.0070 | 0.010 | 0.44 |
| + 3.3 μg ml$^{-1}$ VRP | (0.0010) | (0.002) | (0.16) |
| Sensitisation | 11.0 | 6.1[b] | 8.8 |
| ratio[a] | (0.9) | (1.2) | (1.5) |

[a]Sensitisation ratio = $\frac{ID_{50} - VRP}{ID_{50} + VRP}$

[b]$P = 0.006$ compared with EMT6/P (2-tailed Student's $t$ test). Values given are means of six determinations with standard errors in parentheses.

Although the EMT6/VRP cells are relatively resistant to VRP, they also accumulate more $^3$H-VRP than the parent cells. This is in striking contrast to data for $^3$H-VRP accumulation in MDR cell lines which are VRP hypersensitive (Cano-Gauci & Riordan, 1987; Warr *et al.*, 1988). In these two studies, $^3$H-VRP accumulation was <10% and 20% of parent line levels in cells with VRP-hypersensitivity. Additionally in our EMT6/AR1.0 cell line, which is 50-fold resistant to ADM compared with the EMT6/P parent, there is a modest resistance to VRP cytotoxicity at the same time as a small reduction in $^3$H-VRP accumulation (Reeve *et al.*, 1989). These results taken together apparently indicate that VRP sensitivity is inversely proportional to VRP accumulation. While it is difficult to propose a mechanistic basis for such an inverse relationship, it is clear that cellular accumulation is not the main determinant of VRP sensitivity. This is in contrast to the situation for MDR cell lines where resistance is almost invariably associated with reduced accumulation of MDR type drugs (Bradley *et al.*, 1988).

Resistance to VRP alone is accompanied by a small reduction in the ability of cells to be sensitised to ADM by VRP (from 11.0-fold to 6.1-fold). In previous studies (unpublished) of the sensitisation of EMT6/P cells to ADM at different VRP doses, we have found that a sensitisation ratio of 8.3 at 3.3 μg ml$^{-1}$ of VRP was reduced to 5.5 at 1.65 μg ml$^{-1}$ of VRP. It is clear therefore that a 4-fold reduction in sensitivity to VRP alone is accompanied by a similar reduction in VRP sensitisation to ADM produced by a 2-fold reduction in VRP dose. The biochemical targets for the two processes may therefore be overlapping but more detailed dose–response data will be needed to ascertain whether or not they are identical. Furthermore, as the VRP resistant cells accumulate more VRP than the parent line, it is clear that intercellular VRP concentration is not the determining factor for ADM sensitisation and possibly therefore that an internal domain of P-glycoprotein is not the relevant site for such sensitisation.

The above analysis assumes that the accumulation of $^3$H-VRP reflects the intracellular concentration of the agent. This is by analogy to the intracellular accumulation of agents such as $^3$H-DNR. If, however, $^3$H-VRP is being irreversibly accumulated on the outside of the cell membrane, then analysis of the data becomes more complicated and a variety of alternative approaches become possible.

We will in the future examine in more detail the location of bound $^3$H-VRP in the three cell types described in this paper. This will include measurement of $^3$H-VRP binding to isolated plasma membranes and to TCA-precipitated high molecular weight material. Such studies should allow further elucidation of the relationship between VRP sensitivity, VRP sensitisation to MDR type drugs and the biochemical determinants of the MDR phenotype.

We are grateful to Dr P.H. Rabbitts for carrying out the Northern blot analysis.

### References

BRADLEY, G., JURANKA, P.F. & LING, V. (1988). Mechanism of multidrug resistance. *Biochim. Biophys. Acta*, **948**, 87.

CANO-GAUCI, D.F. & RIORDAN, J.R. (1987). Action of calcium antagonists on multidrug resistant cells: specific cytotoxicity independent of increased cancer drug accumulation. *Biochem. Pharmacol.*, **36**, 2115.

COLEY, H.M., TWENTYMAN, P.R. & WORKMAN, P. (1989*a*). Identification of anthracyclines and related agents which retain preferential activity over adriamycin in multidrug resistant cell lines, and further resistance modification by verapamil and cyclosporin A. *Cancer Chemother. Pharmacol.*, **24**, 284.

COLEY, H.M., TWENTYMAN, P.R. & WORKMAN, P. (1989*b*). Improved cellular accumulation is characteristic of anthracyclines which retain high activity in multidrug resistant cell lines, alone or in combination with verapamil or cyclosporin A. *Biochem. Pharmacol.* (in the press).

CORNWELL, M.M., PASTAN, I. & GOTTESMAN, M.M. (1987). Certain calcium channel blockers bind specifically to multidrug-resistant human KB carcinoma membrane vesicles and inhibit drug binding to P-glycoprotein. *J. Biol. Chem.*, **262**, 2166.

DENHARDT, D.T. (1966). A membrane-filter technique for detection of complementary DNA. *Biochem. Biophys. Res. Commun.*, **23**, 641.

FEINBERG, A.P. & VOGELSTEIN, B. (1984). A technique for radiolabelling DNA restriction endonuclease fragments to high specific activity. *Anal. Biochem.*, **137**, 266.

FOJO, A., AKIYAMA, S., GOTTESMAN, M.M. & PASTAN, I. (1985). Reduced drug accumulation in multiply drug-resistant human KB carcinoma cells lines. *Cancer Res.*, **45**, 3002.

GROS, P., CROOP, J. & HOUSMAN, D. (1986). Mammalian multidrug resistance gene: complete cDNA sequence indicates strong homology to bacterial transport proteins. *Cell*, **47**, 371.

MOSMANN, T. (1983). Rapid colorimetric assay for cellular growth and survival: application to proliferation and cytotoxicity assays. *J. Immunol. Methods*, **65**, 55.

REEVE, J.G., WRIGHT, K.A., RABBITTS, P.H., TWENTYMAN, P.R. & KOCH, G.L.E. (1989). Collateral resistance to verapamil in multidrug resistant mouse tumor cells. *J. Natl Cancer Inst.*, **81**, 588.

SLATER, L.M., MURRAY, S.L. & WETZEL, M.W. (1982). Verapamil restoration of daunorubicin responsiveness in daunorubicin-resistant Ehrlich ascites carcinoma. *J. Clin. Invest.*, **70**, 1131.

TAYLOR, I.W. (1980). A rapid single step staining technique for DNA analysis by flow micro-fluorimetry. *J. Histochem. Cytochem.*, **28**, 1021.

THOMAS, P.S. (1980). Hybridisation of denatured RNA and small DNA fragments transferred to nitrocellulose. *Proc. Natl Acad. Sci. USA*, **77**, 5201.

TSURUO, T., IIDA, H., TSUKAGOSHI, S. & SAKURAI, Y. (1981). Overcoming of vincristine resistance in P388 leukaemia in vivo and in vitro through enhanced cytotoxicity of vincristine and vinblastine by verapamil. *Cancer Res.*, **41**, 1967.

TSURUO, T., IIDA, H., TSUKAGOSHI, S. & SAKURAI, Y. (1982). Increased accumulation of vincristine and adriamycin in drug-resistant tumor cells following incubation with calcium antagonists and calmodulin inhibitors. *Cancer Res.*, **42**, 4730.

TSURUO, T., IIDA, H., TSUKAGOSHI, S. & SAKURAI, Y. (1983). Potentiation of vincristine and adriamycin effects in human hemopoietic tumor cell lines by calcium antagonists and calmodulin inhibitors. *Cancer Res.*, **43**, 2267.

TWENTYMAN, P.R., FOX, N.E. & BLEEHEN, N.M. (1986*a*). Drug resistance in human lung cancer lines: cross-resistance studies and effects of the calcium transport blocker, verapamil. *Int. J. Radiat. Oncol. Biol. Phys.*, **12**, 1355.

TWENTYMAN, P.R., FOX, N.E., WRIGHT, K.A. & 4 others (1986*b*). The *in vitro* effects and cross-resistance patterns of some novel authracyclines. *Br. J. Cancer*, **53**, 585.

TWENTYMAN, P.R. & LUSCOMBE, M. (1987). A study of some variables in a tetrazolium dye (MTT) based assay for cell growth and chemosensitivity. *Br. J. Cancer*, **56**, 279.

WARR, J.R., ANDERSON, M. & FERGUSSON, J. (1988). Properties of verapamil-hypersensitive multidrug-resistant Chinese hamster cell lines. *Cancer Res.*, **48**, 4477.

WARR, J.R., BREWER, F., ANDERSON, M. & FERGUSSON, J. (1986). Verapamil hypersensitivity of vincristine resistant Chinese hamster ovary cell lines. *Cell Biol. Int. Rep.*, **10**, 389.

WATSON, J.V. (1980). Enzyme kinetic studies in cell populations using fluorogenic substrates and flow cytometric techniques. *Cytometry*, **1**, 143.